# Inflammatory Responses Potentiate GAS M Protein Induced Cardiac Damage in an Experimental Model of Rheumatic Heart Disease

**DOI:** 10.1002/iid3.70221

**Published:** 2025-07-11

**Authors:** Rukshan A. M. Rafeek, Simone L. Reynolds, Manisha Pandey, David J. McMillan, Kadaba S. Sriprakash, Michael F. Good, Natkunam Ketheesan

**Affiliations:** ^1^ School of Science & Technology University of New England Armidale New South Wales Australia; ^2^ Institute for Biomedicine and Glycomics Griffith University Brisbane Queensland Australia; ^3^ QIMR Berghofer Medical Research Institute Brisbane Queensland Australia; ^4^ School of Science, Technology and Engineering University of the Sunshine Coast Brisbane Queensland Australia

**Keywords:** acute rheumatic fever, animal model, pertussis toxin, rheumatic heart disease

## Abstract

**Aim:**

Acute rheumatic fever (ARF) and Rheumatic heart disease (RHD) are autoimmune sequalae, that develops in a proportion of individuals exposed to group A streptococcal infection. The autoimmune pathology of ARF/RHD is multifactorial. Both host and pathogen‐associated factors including genetic predisposition, inflammatory responses, tissue cross‐reactive antibodies and T‐cells contribute to disease development and progression. Hitherto, the role of inflammatory responses in ARF/RHD has never been demonstrated in animal models. In this study for the first time, using the Rat Autoimmune valvulitis model of RHD, we demonstrate the requirement for inflammatory responses in promoting cardiac damage in ARF/RHD.

**Methods:**

To determine the role of inflammatory responses we used *Bordetella pertussis* toxin (BPTx) as an adjuvant to enhance the inflammatory responses initiated by GAS M protein.

**Results:**

Lewis rats injected with GAS rM5 emulsified in Complete Freund's Adjuvant (CFA) and co‐adjuvant BPTx, had enhanced valvulitis and inflammatory changes as shown by; (a) elevated levels of circulating inflammatory cytokines; (b) functional changes characterized by a prolonged P‐R interval in electrocardiography, (c) enhanced cross‐reactive antibody production against cardiac and connective tissue proteins; and (d) increased infiltration of IFN‐γ+ and IL‐17A+ secreting leukocytes into myocardium and valvular tissues.

**Conclusion:**

These studies have established that in addition to exposure to GAS M protein, BPTx accelerate the inflammatory and autoimmune processes leading to cardiac damage. These observations substantiate the hypothesis that, in susceptible individuals robust inflammatory responses facilitate the progression of ARF/RHD.

## Graphical abstract

Group A streptococcal M protein elicit antibody and T‐cell responses that cross react with cardiac and connective tissue proteins in the Rat Autoimmune Valvulitis (RAV) model of rheumatic heart disease. Bordetella pertussis Toxin induce a robust Th1 response that potentiates the autoimmune process in the RAV model A robust Th1/Th17 response was found to be a major driver of cardiac pathology in the RAV model

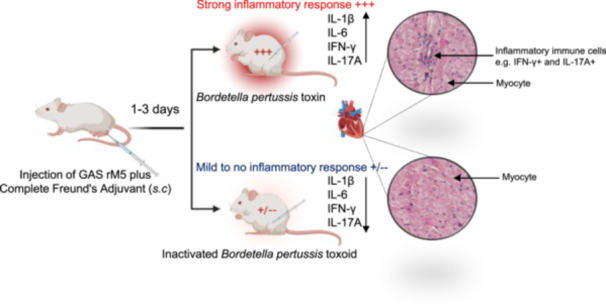

**Abbreviations**
ARF: Acute rheumatic fever
BPToxoid: *Bordetella Pertussis* whole cell antigen extract
BPTx: *Bordetella pertussis* toxin
CFA: Complete freund's adjuvant
EAE: experimental autoimmune encephalitis
EAMG: experimental autoimmune myasthenia gravis
EAU: experimental autoimmune uveoretinitis
GAS: Group A streptococcus
IFA: Incomplete freund's adjuvant
i.p.: intraperitoneal
LPS: Lipopolysaccharide
PBS: Phosphate buffered saline
RAV: Rat autoimmune valvulitis model
RHD: Rheumatic heart disease
*s.c*.: subcutaneous

## 1 Introduction

Acute rheumatic fever (ARF) is a multiorgan disorder triggered by group A streptococcal (GAS) infection in susceptible individuals. One or more episodes of ARF can lead to Rheumatic heart disease (RHD) resulting in severe damage to heart valves [1]. ARF/RHD is an autoimmune disease induced by antibodies and T‐cells directed against GAS antigens [2]. Anti‐GAS antibodies target the cardiac endothelium, enables infiltration of T‐cells and facilitates inflammation of both the heart valves and the myocardium. Multiple lines of evidence also suggests that repeated GAS infections prime pro‐inflammatory cytokines such as IL‐6, TNF‐α, IL‐8/CXCL8, IFN‐γ, and IL‐17A and play a critical role in driving the inflammatory process leading to irreversible cardiac tissue damage [3, 4, 5]. In addition, variations in some MHC Class II molecules may be associated with aberrant T‐cell activation [6]. This combination could lead to increased production of inflammatory cytokines and cross‐reactive antibodies against cardiac proteins, which facilitates heart damage [7, 8, 9]. The contribution of inflammatory responses in promoting cardiac damage in ARF/RHD, has never been experimentally demonstrated in a preclinical model. Experimental models are invaluable to investigate the complex pathology observed in autoimmune disorders. The advent of the rat autoimmune valvulitis (RAV) model developed by Quinn et al. [10] and characterized by our group is considered the most credible animal model to investigate the pathogenesis of ARF/RHD [11, 12, 13, 14, 15, 16, 17, 18, 19, 20, 21, 22, 23, 24].

In the RAV model, salient clinical features of ARF/RHD can be induced, without the need of GAS infection. This can be achieved by subcutaneous (s.c.) injection of whole killed GAS or GAS recombinant M‐proteins (rM) emulsified in Complete Freund's Adjuvant (CFA) followed by *Bordetella pertussis* toxin (BPTx) and subsequent booster doses of GAS rM5 protein in Incomplete Freund's Adjuvant (IFA). CFA and IFA are two commonly used adjuvants, known to enhance the immune response and direct the specificity of antigen recognition [25]. Adjuvants are widely used to induce autoimmune diseases in rodents. In experimental models, adjuvants have been shown to enhance both innate and adaptive immune responses [26], while recapitulating the immune activation pathways elicited by infectious agents required for the induction of autoimmune pathology [27]. In addition to CFA and IFA, BPTx is used as a co‐adjuvant to boost the adjuvant activity of CFA, and has been shown to enhance immune responses to co‐administered antigens [28]. The recruitment of BPTx as a co‐adjuvant is a well‐established strategy employed in animal models of experimental autoimmune encephalitis (EAE) [28], experimental autoimmune uveoretinitis (EAU) [29] experimental autoimmune myasthenia gravis (EAMG) [30] and RAV [11, 12, 13, 14, 24, 31, 32]. BPTx is an exotoxin produced by *Bordetella pertussis* and has multiple effects on the immune system. However, the exact mechanism of action of BPTx in rodent models of autoimmune disease, including the RAV model, remains unclear. Studies suggest that the administration of BPTx skews the immune response towards Th1/Th17 pathways [33], priming the system for a pro‐inflammatory cytokine response and robust antibody response to circulating antigens [34, 35]. By comparing the humoral and cellular responses induced by the administration of BPTx versus *Bordetella pertussis* whole cell antigen extract following injection of GAS M‐protein, we demonstrate that BPTx induces the inflammatory conditions that accelerate the development of GAS rM5 mediated cardiac damage, reflecting the repetitive inflammatory challenges observed in patients with ARF/RHD. Reproducing the immunopathological features of infection this study provides insight as to why only a small proportion (0.3%–3%) of individuals who acquire GAS skin or pharyngeal infection go on to develop ARF/RHD [36, 37].

## 2 Materials and Methods

### 2.1 Animals

All experimental protocols involving animals were approved by the Animal Ethics Committee of University of New England (UNE) (AEC 19‐013/ARA22‐065). All procedures involving animals were in accordance with Australian code for the care and use of animals for scientific purposes and followed the ARRIVE guidelines. Female Lewis rats (LEW/SsN; Albino:a,h,c:RT^1^) aged 4 to 6 weeks were purchased from the Centre for Animal Research and Teaching, UNE, Australia. A sample size of ≥ 4 per group was considered adequate to achieve statistical power, based on our previous findings [11, 12, 13, 14, 15, 16, 17, 18, 19, 20, 21, 22, 23, 24]. All rats were housed in individually ventilated cages with a maximum of 3–4 rats/cage and were acclimatized for 5 days before experiments. The relative humidity of housing unit was ranged between 45% and 65% and temperature at 20°C–22°C and rats were exposed to a 12‐h light–dark cycle.

### 2.2 Preparation of GAS M Proteins

Recombinant M5 protein of GAS (rM5) was cloned and purified as described previously [20]. Briefly, GAS *emm5* was cloned into pREP4 vector and expressed in *E. coli* BL21. Recombinant M proteins were purified using Ni‐NTA resin. Lipopolysaccharide (LPS) contamination in recombinant protein preparations was removed by Triton X‐114 LPS extraction as previously described [38].

### 2.3 Adjuvants

Adjuvants used in this study were *Bordetella pertussis* whole cell antigen extract (BPToxoid; Alpha Diagnostic International, USA), which contains formalin inactivated pertussis toxin, filamentous hemagglutinin and whole‐cell antigen and BPTx (Gibco, USA), a purified exotoxin secreted by *Bordetella pertussis*. BPTx contains both subunit A and B. Subunit A has ADP‐ribosyltransferase enzymatic activity, which interferes with G protein‐dependent signal transduction. Subunit B is associated with cell surface binding specificity of the toxin and delivery of subunit A into the cells [39]. CFA and IFA were purchased from Sigma Australia.

### 2.4 Induction of Autoimmune Carditis and Valvulitis

**Experiment 1:** Twenty age‐matched female Lewis rats were randomly divided into three groups (*n* = 5); (i) Group (A): PBS/CFA/0.6 µg BPTx (control), (ii) Group (B): GAS rM5/CFA/0.6 µg BPTx, (iii) Group (C): GAS rM5/CFA/0.6 µg BPToxoid.

**Experiment 2:** In a separate experiment sixteen age‐matched female Lewis rats were divided into four treatment groups (*n* = 4); (i) Group (A): PBS/CFA/0.6 µg BPTx (control), (ii) Group (B): GAS rM5/CFA/0.6 µg BPTx, (iii) Group (C): GAS rM5/CFA/0.3 µg BPTx, (iv) Group (D): GAS rM5/CFA/0.0 µg BPTx.

Baseline electrocardiography (ECG) was performed before subcutaneous (s.c.) injection of 0.5 mg/100 μL of recombinant GAS M5 protein emulsified in CFA in the hock as described previously [21]. Control rats were injected with PBS emulsified in CFA. All priming injections were performed under isoflurane inhalation anaesthesia (5% induction and 2% maintenance).

In experiment 1, on day 1 and 3 all rats in group A and group B were intraperitoneally (i.p.) injected with 0.6 µg of BPTx whereas rats in group C were injected with BPToxoid (Alpha Diagnostic International, USA) in 200 μL of PBS. In experiment 2, rats in group A and B were injected with 0.6 µg BPTx and rats in group C were injected with 0.3 µg BPTx. On days 7, 14, 21, and 28 all rats were boosted with respective antigens in IFA on the flank (Supplementary Figure 1A). The rats were euthanized on day 35 from the day of primary injection with the overdose of sodium pentobarbital (260 mg/kg, i.p), blood was collected by cardiac puncture and heart tissue was collected and fixed with 4% paraformaldehyde for histopathological analysis. Spleen tissue was collected in RPMI+ Penicillin and Streptomycin for the isolation of splenocytes. Myocarditis and valvulitis were determined based on the following criteria: (1) Changes in P‐R interval in ECG; (2) Elevated levels of tissue cross‐reactive antibodies; (2) Varying degrees of mononuclear cell infiltration into cardiac tissue.

### 2.5 Electrocardiography (ECG)

ECG traces were recorded for 1–2 min using Bio Amp with PowerLab data acquisition system (ADInstruments, USA) in all rats before injection and a day before euthanasia to assess the conduction abnormalities in the heart [16].

### 2.6 Splenocytes Culture and Flow Cytometry

A single‐cell suspension of splenocytes was prepared and splenic red blood cells were lysed using ammonium‐chloride‐potassium lysis buffer (ACK buffer). 1 × 10^6^ splenocytes per sample were cultured in RPMI supplemented with 10% FBS, 1% glutamax, and 1% pen‐strep at 37°C and 5% CO_2_ with GAS rM5 protein and Concanavalin A (positive control) or media (negative control) for 66 h as described elsewhere [12, 13, 14]. Brefeldin A (Biolegend, USA) was added to the wells at 10 μg/mL, and cells were incubated for an additional 6 h. After 72 h of incubation, the supernatant was collected to assess the levels of inflammatory cytokines including IL‐1β, IL‐6, IFN‐γ, and IL‐17A. Cells were collected for Fluorescence‐activated cell sorting (FACS). Cells were washed and stained in v‐bottom 96 well plate, then incubated with Zombie B550™ Fixable Viability Dye (Biolegend, USA) to discriminate between viable and dead cells. Cells were stained with surface antibodies against CD3^+^ and CD4^+^ (Biolegend, USA) for 30 min at 4°C. Following fixation and permeabilization cells were incubated with intracellular antibodies against IFN‐γ before being analyzed through BD Accuri C6 plus (BD Biosciences, USA).

### 2.7 ELISA to Detect Inflammatory Cytokines and Serum Antibody

Inflammatory cytokines IL‐1β (Bioss Inc, USA), IL‐6 (LEGEND MAX™ Rat IL‐6 ELISA Kit), IFN‐γ (LEGEND MAX™ Rat IFN‐γ ELISA Kit), and IL‐17A (ELISA MAX™ Deluxe Set Rat IL‐17A) in serum were analyzed using ELISA kits from Biolegend USA according to the manufacturer's instructions.

Serum antibody reactivity against BPTx, BPToxoid and purified host proteins including cardiac myosin, tropomyosin, laminin, and keratin (Sigma, USA) were determined using indirect ELISA as described previously [17]. Briefly 100 μL of antigens (10 μg mL^−1^ of cardiac myosin, tropomyosin, laminin, keratin, BPTx and BPToxoid) were coated onto Maxisorp 96‐well plates (Nunc, USA). Plates were washed and blocked with 1% BSA and individual rat sera were added in duplicate at 1:400 dilution. Following repeated washing of the plates, each well was incubated with 100 µL of goat anti‐rat IgG HRP conjugated secondary antibody (1:5000; Jackson Immunoresearch, USA) for an hour at room temperature. Each well was washed four times with 150 µL of wash buffer. The plate was dried and 100 µL of 2‐2′–azino‐di (3‐ethylbenzthiazoline)‐6‐sulphonate (ABTS) (Sigma, USA) was added to each well and incubated for 20 min. Absorbance was measured at 415 nm using SpectraMax M2/M2e (Molecular Devices, USA).

### 2.8 Histology and Immunohistochemistry of Cardiac Tissue

To examine the extent of inflammation, formalin‐fixed cardiac tissue was processed, embedded in paraffin, sectioned, and stained with Harris H&E using standard procedures as previously described [20]. These sections were scored using a validated semi‐quantitative scoring system [24]. Masson's trichrome staining was performed to observe deposition of collagen fibres in the mitral valve and myocardium following standard procedures. The percentage of collagen deposition in the myocardium and valvular tissue was determined using the ImageJ software®. A minimum of five photographed areas of myocardium were used to analyse data.

Formalin‐fixed, paraffin‐embedded sections (FFPE, 5 µm) were deparaffinised in xylene and rehydrated with graded ethanol. Epitope retrieval was done with 10 mM Tris‐EDTA (pH 9.0) for 15 min at 700 W in a laboratory microwave. Sections were incubated in 3% H_2_O_2_ for 10 min to inhibit endogenous peroxidase activity and rinsed in 10 mM Tris Buffered Saline (TBS) at pH 7.4 three times for 5 min. These were then blocked with 10% normal sheep serum in TBS for an hour. Blocking buffer was removed and incubated with rat anti‐CD3^+^ (Abcam, USA), rat anti‐CD68^+^ (Abcam, USA), rat anti‐IFN‐γ^+^ (Bioss Inc, USA), rat anti‐IL‐17A^+^ (Abcam, USA), and mouse anti‐rat CD106 (VCAM‐1, Bio‐rad, USA) at 4°C overnight in a humidified chamber. Sections were washed in TBS and incubated with biotinylated goat anti‐rat IgG (Jackson Immunoresearch, USA) diluted at 1:500 for 2 h at room temperature in a humidified atmosphere. After rinsing in TBS, sections were incubated with avidin‐biotin Complex (VECTASTAIN® Elite ABC‐Peroxidase Kit) for an hour at room temperature in a humidified environment. Sections were rinsed three times in TBS and developed using 3, 3’‐ diaminobenzidine (DAB) substrate (Sigma, Australia) and counter stained with Harris Haematoxylin.

### 2.9 Scoring of Tissue Infiltrating CD3^+^, CD68^+^, IL‐17A^+^, IFN‐Γ^+^ and CD106^+^ Cells

The degree of infiltration of CD3^+^, CD68^+^, IL‐17A^+^, and IFN‐γ^+^ were scored based on a 4‐grade scale: grade 0, no infiltration; grade 1, infiltration of 13 cells; grade 2, infiltration of 3–5 cells; and grade 3, infiltration of > 5 cells (Supplementary Table 1). Image analysis and acquisition was performed using a Hamamatsu NanoZoomer 2.0 slide scanner (Hamamatsu, San Jose, CA), NDP. view2 Plus. The percentage of expression of CD106 (VCAM‐1) in the myocardium was determined on the digital images using the ImageJ software®. A minimum of three photographed areas of myocardium were used to analyse the data [14].

### 2.10 Statistical Analysis

ECG, histology score, and absorbance (OD values) were assessed using GraphPad Prism 8 (GraphPad, USA). All data from experimental and control groups passed D'Agostino & Pearson omnibus normality tests. Tests of significance of the ELISA data were analyzed using Two‐way analysis of variance (ANOVA) with Tukey's post hoc multiple comparisons test. Non‐parametric data were analyzed using Mann‐Whitney *U* test, *p* values less than 0.05 was considered significant.

## 3 Results

### 3.1 BPTx Induces a Robust Inflammatory Response to GAS rM5

**Figure 1:**
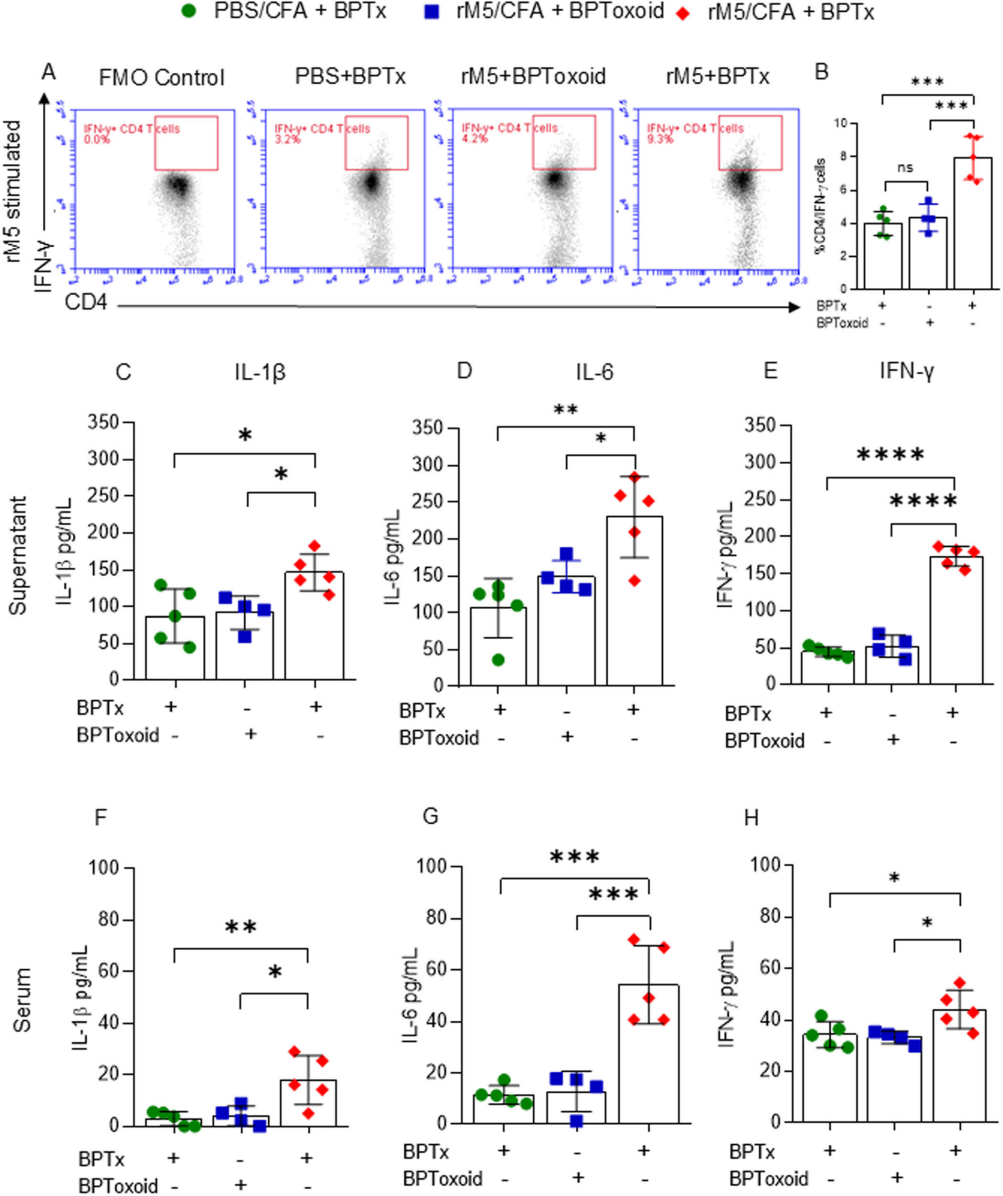
BPTx promotes development of a Th1 response. BPTx upregulates antigen specific IFN‐γ production. Splenocytes were harvested 35 days after initial and three booster injection with GAS rM5 and re‐stimulated with GAS rM5. IFN‐γ producing T‐cells were assessed by Flow cytometry and the production of IL‐1β, IL‐6 and IFN‐γ in 72‐h culture supernatants and serum were determined by ELISA. Significantly higher levels of IFN‐γ producing CD4^+^ T‐cells were observed in splenocytes from rats injected with GAS rM5/CFA/BPTx re‐stimulated with GAS rM5 compared to spleen cells from GAS rM5/CFA/BPToxoid and PBS rM5/CFA/BPTx (A and B). There are elevated levels of IL‐1β, IL‐6, and IFN‐γ in supernatants from cultured splenocytes (C–E) and serum (F–H) in rats injected with GAS rM5/CFA/BPTx compared to GAS rM5/CFA/BPToxoid and PBS/CFA/BPTx. Data are from single experiment with age‐matched rats injected with GAS rM5 (*n* = 4/5). Statistics: mean ± SEM (*n* = 5); (B–D) One‐way ANOVA with Tukey's post hoc multiple comparison test; *p < 0.05, **p < 0.01 and ***p < 0.001.

Several studies have shown that T helper cell cytokines, including IFN‐γ^+^ and IL‐17A^+^ play a major role in driving the pathogenesis of ARF/RHD in both humans and rodents. Hence, we initially analysed IFN‐γ producing T‐cells by FACS upon *ex vivo* restimulation of rat splenocytes with antigen GAS rM5. The number of IFN‐γ producing T‐cells was significantly higher in re‐stimulated splenocytes from rats injected with GAS rM5 in combination with BPTx (GAS rM5/CFA/BPTx) compared to the rats that received GAS rM5 with inactive toxin (GAS rM5/CFA/BPToxoid) and rats injected with PBS (PBS/CFA/BPTx) (Figure 1A). Furthermore, significantly elevated levels of IL‐1β, IL‐6, and IFN‐γ were observed in the serum and supernatant of splenocytes re‐stimulated with GAS rM5 from rats injected with GAS rM5/CFA/BPTx compared to GAS rM5/CFA/BPToxoid or PBS/CFA/BPTx (Figure 1C–H). Interestingly, despite the presence of GAS rM5 in the BPToxoid injected group, the percentage of CD4/IFN‐ɣ+ cells were not significantly different to those seen in rats injected with BPTx alone group (Figure 1B). Additionally, the cytokine levels in the supernatant of the splenocytes and the serum in both these groups were comparable.

### 3.2 GAS Antigen Induced Inflammatory Changes in Cardiac Tissue Are Driven by BPTx

**Figure 2:**
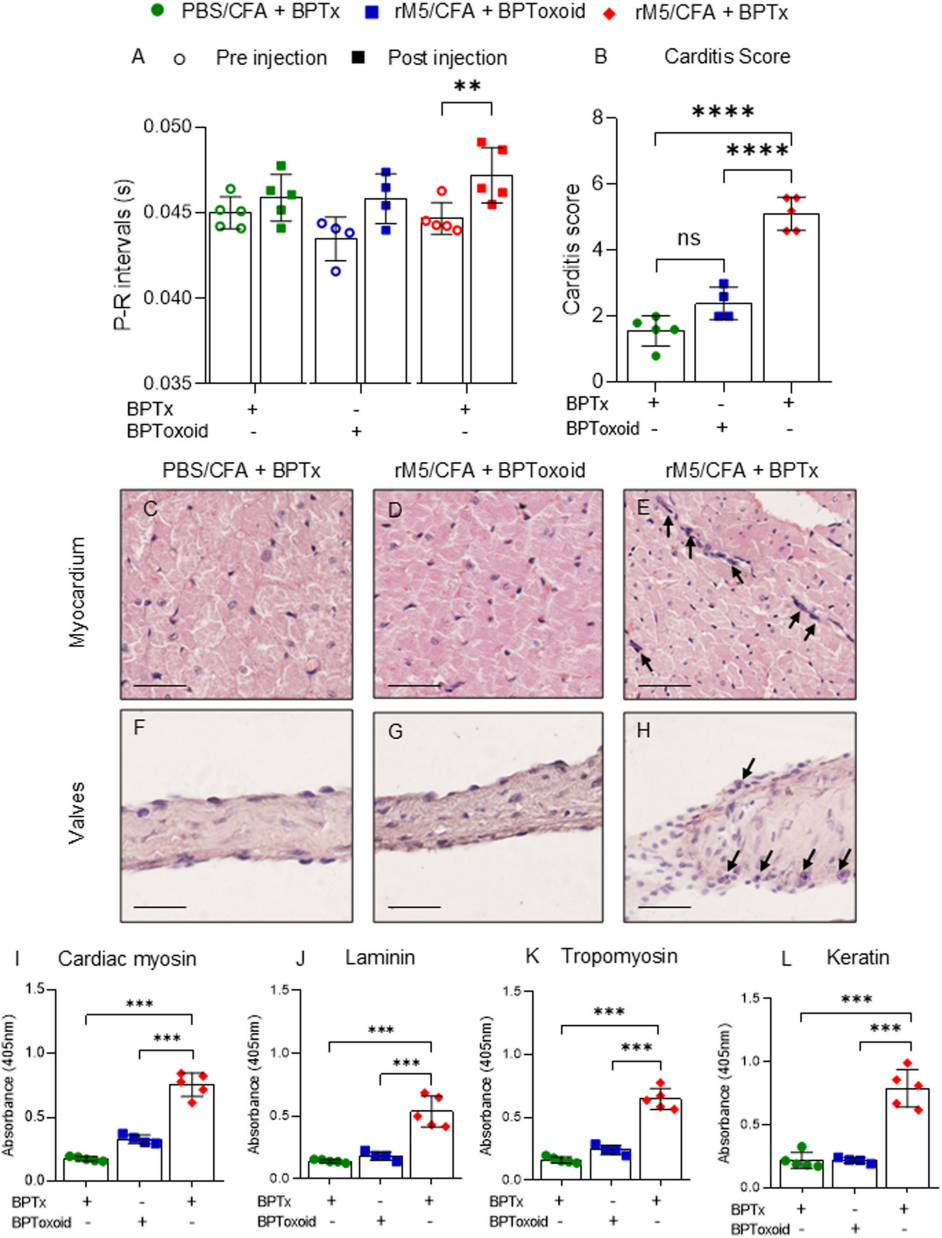
BPTx induced responses lead to cardiac functional impairment, increased inflammation and production of tissue cross‐reactive antibodies. ECG was performed pre and post GAS rM5 injection to assess conduction abnormalities or functional changes in the heart. Rats injected with GAS rM5/CFA/BPTx showed prolonged P‐R interval compared to pre‐injection. No ECG changes were observed pre and post injection of GAS rM5/CFA/BPToxoid and PBS/CFA/BPTx (A). Cardiac tissues were stained with H&E to assess the infiltration of mononuclear cells. Significantly higher infiltration with mononuclear cells were observed in the myocardium and the valvular tissues of rats injected with GAS rM5/CFA/BPTx (E and H) compared to GAS rM5/CFA/BPToxoid (D and G) and PBS/CFA/BPTx (C and F). Carditis cores (B) which quantify mononuclear cell infiltration were significantly higher in tissues from rats injected with GAS rM5/CFA/BPTx (E, H) compared to GAS rM5/CFA/BPToxoid (D and G) and PBS/CFA/BPTx (C and F). Furthermore, elevated levels of cross‐reactive antibodies against cardiac myosin (I), laminin (J), tropomyosin (K), and keratin (L) were observed in serum from rats injected with GAS rM5/CFA/BPTx (E and H) compared to GAS rM5/CFA/BPToxoid. Images magnified at ×200 and scale bar represent 50 µm. Data are from single experiment with age‐matched rats injected with GAS rM5 (*n* = 4/5). Statistics: mean ± SEM (*n* = 5); (A, I–L) One‐way ANOVA with Tukey's post hoc multiple comparison test; (B) *t* test; **p < 0.01, ***p < 0.001 and ****p < 0.0001.

Conduction abnormalities of cardiac tissue are key features in ARF/RHD and characterized by prolongation of the P‐R interval in ECG. The P‐R intervals in rats, within each group, were assessed before and after injection using ECG. Rats injected with GAS rM5/CFA/BPTx showed a significantly prolonged P‐R interval compared to rats injected with GAS rM5/CFA/BPToxoid or PBS/CFA/BPTx (Figure 2A). Carditis scores were assessed based on the number of infiltrating mononuclear cells in both the myocardium and valves. Cell infiltration into cardiac tissue was significantly higher in rats injected with GAS rM5/CFA/BPTx than rats injected with GAS rM5/CFA/BPToxoid (Figure 2B). Histological examination of the heart tissue of rats injected with GAS rM5/CFA/BPToxoid and PBS/CFA/BPTx showed little or no evidence of inflammation (Figure 2C,D,F,G). Whereas cardiac tissue of rats injected with GAS rM5/CFA/BPTx demonstrated significantly higher infiltration of mononuclear cells (Fig. E, H). To confirm the drivers of mononuclear cell infiltration, we assessed the levels of tissue cross‐reactive antibodies in serum. The presence of antibodies to the M‐protein (Supplementary Figure 1C,D), that cross‐react with host tissue proteins is also a hallmark of ARF/RHD. Rats injected with GAS rM5/CFA/BPTx developed significantly elevated levels of antibodies reactive against the tissue proteins cardiac myosin, laminin, tropomyosin, and keratin compared to rats injected with GAS rM5/CFA/BPToxoid or PBS/CFA/BPTx (Figure 2I–L). Furthermore, rats injected with BPToxoid and BPTx develop significant antibody levels to their corresponding antigens and low levels of antibody reactivity are observed against BPTx and BPToxoid, respectively (Supplementary Figure 1B).

### 3.3 Effect of Escalating Doses of BPTx in the Induction of Carditis

**Figure 3:**
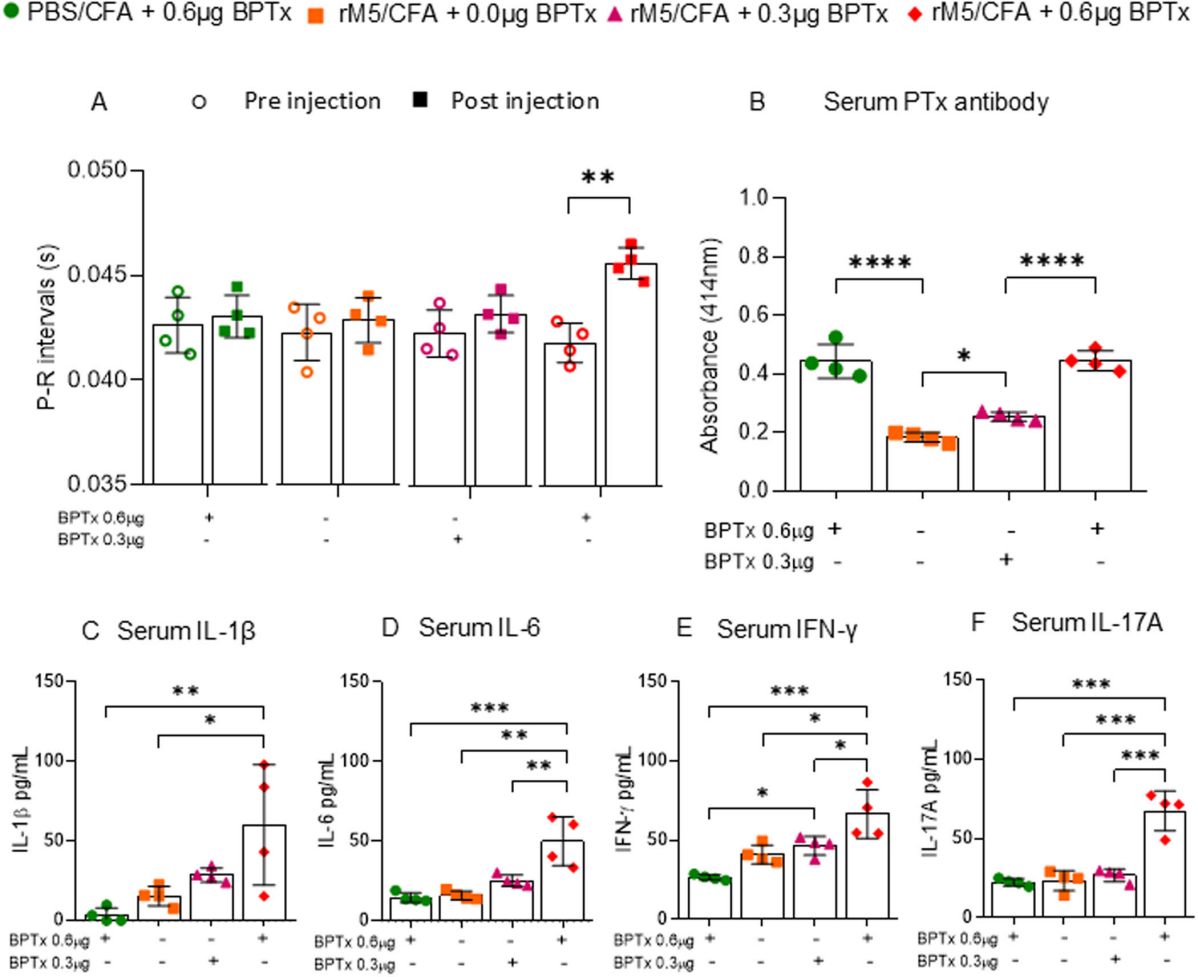
BPTx is required for the induction of inflammatory responses. ECG was performed pre and post injection of GAS rM5/CFA and PBS/CFA and two different concentration (0.3 and 0.6 µg) of BPTx, to determine the minimal concentration of BPTx required to induce conduction abnormalities in the heart. Rats injected with GAS rM5/CFA/0.6 µg BPTx showed prolonged P‐R interval compared to rats injected with GAS rM5/CFA/0.3 µg BPTx and GAS rM5/CFA alone or PBS/CFA/0.6 µg BPTx (A). Levels of anti‐BPTx antibodies after injection of rats with GAS rM5/CFA/0.6 µg BPTx, GAS rM5/CFA/0.3 µg BPTx, GAS rM5/CFA, and PBS/CFA/0.6 µg BPTx (B). We also analyzed key cytokines circulating in the serum. Significantly higher levels of circulating IL‐1β (C), IL‐6 (D), IFN‐γ (E), and IL‐17A (F) in rats injected with GAS rM5/CFA/0.6 µg BPTx. This group also showed prolonged P‐R interval compared to rats injected with GAS rM5/CFA/0.3 µg BPTx, GAS rM5/CFA alone or PBS/CFA/0.6 µg BPTx. Data are from single experiment with age‐matched rats injected with GAS rM5 (*n* = 4). Statistics: mean ± SEM (*n* = 4); (A,C–F) One‐way ANOVA with Tukey's post hoc multiple comparison test; (B) *t* test; *p < 0.05, **p < 0.01, ***p < 0.001 and ****p < 0.0001.

Inflammation plays a key role in the pathogenesis of ARF/RHD, we therefore investigated the minimal dose of BPTx required in the RAV model, to induce inflammatory changes in cardiac tissue. We explored administration of BPTx at two concentrations; 0.3 and 0.6 µg, in combination with GAS rM5 or without GAS rM5 (PBS control). Rats developed a BPTx‐specific antibody response, and we observed that with the increasing concentration of BPTx the levels of anti‐BPTx antibodies also increased (Figure 3B). The presence or absence of GAS rM5 appeared to have no impact on the generation of BPTx‐specific antibodies, with no significant difference observed between the two groups that received 0.6 µg of BPTx (GAS rM5/CFA/0.6 µg BPTx and PBS/CFA/0.6 µg BPTx). Interestingly, we found that significant functional changes, characterized by prolonged P‐R interval in ECG, were only observed in the GAS rM5/CFA/0.6 µg BPTx group. (Figure 3A). This finding was in line with the inflammatory changes observed in each group. Here, we also observed elevated levels of the inflammatory cytokines such as IL‐1β, IL‐6, IFN‐ γ and IL‐17A, in the serum of rats injected with GAS rM5/CFA/0.6 µg BPTx compared to rats injected with GAS rM5/CFA/0.3 µg BPTx or GAS rM5/CFA or PBS/CFA/0.6 µg BPTx (Figure 3C–F).

### 3.4 BPTx Enhances Inflammatory Changes, Collagen Deposition and Tissue Cross‐Reactive Antibody Responses

**Figure 4:**
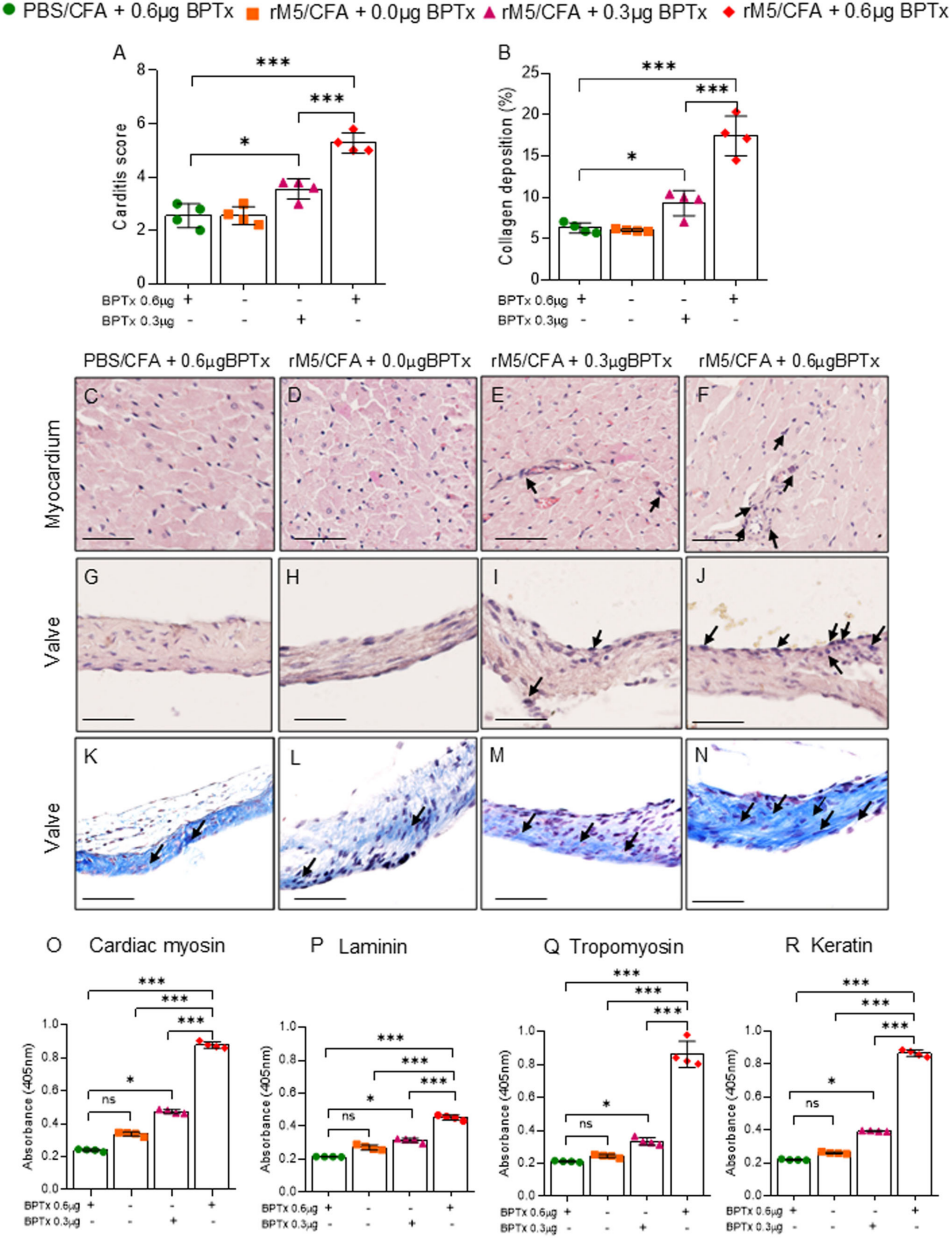
BPTx induced inflammatory changes lead to collagen deposition and production of host proteins cross‐reactive antibodies. Mononuclear cell infiltration into the myocardium and valvular tissue (A) represented by carditis score. The proportion of collagen deposition observed in valvular tissue (B). Significantly higher infiltration of mononuclear cells and collagen deposition were observed in cardiac tissue from rats injected with GAS rM5/CFA/0.6 µg BPTx (F, J, N) compared to rats injected with GAS rM5/CFA/0.3 µg BPTx (E, I, M) and GAS rM5/CFA alone (D, H, L) or PBS/CFA/0.6 µg BPTx (C, G, K). Furthermore, significantly higher levels of cross‐reactive antibodies against host proteins cardiac myosin (O), laminin (P), tropomyosin (Q), and keratin (R) were observed serum from rats injected with GAS rM5/CFA/0.6 µg BPTx compared to rats injected with GAS rM5/CFA/0.3 µg BPTx and GAS rM5/CFA alone or PBS/CFA/0.6 µg BPTx. Images magnified at ×200 and scale bar represent 50 µm. Data are from single experiment with age‐matched rats injected with GAS rM5 (*n* = 4). Statistics: mean ± SEM (*n* = 4); (A and B) *t* test; (O–R) One‐way ANOVA with Tukey's post hoc multiple comparison test; ns –not significant, *p < 0.05, **p < 0.01, ***p < 0.001 and ****p < 0.0001.

Carditis and valvulitis were assessed by the presence of infiltrating mononuclear cells in cardiac tissues following hematoxylin and eosin staining. Rats injected with GAS rM5/CFA/0.6 µg BPTx (Figure 4F,J) had an increased infiltration of mononuclear cells in the myocardium and valves. Fewer infiltrating cells were observed in rats injected with GAS rM5/CFA/0.3 µg BPTx (Figure 4E,I), and little or no evidence of inflammatory cell infiltration was observed in rats injected with GAS rM5/CFA alone (Figure 4D,H) or in control rats injected with PBS/CFA/0.6 µg BPTx (Figure 4C,G). Rats injected with GAS rM5/0.6 µg BPTx had significantly higher carditis scores than rats injected with GAS rM5/0.3 µg BPTx or rM5 without BPTx and control rats injected with PBS (Figure 4A). In addition, collagen deposition and fibrosis in the valves was assessed by Masson's trichrome stain. Of note, the proportion of collagen deposition was significantly higher in rats injected with GAS rM5/CFA/0.6 µg BPTx (Figure 4N) compared to rats injected with GAS rM5/0.3 µg BPTx (Figure 4M). No significant difference in collagen deposition was observed in rats injected with GAS rM5/CFA alone and PBS/CFA/BPTx (Figure 4K,L).

In ARF/RHD antibodies against GAS M protein cross‐react with host cardiac and connective tissue proteins and are thought to initiate the inflammatory response in tissues. In the RAV model, antibodies against GAS rM5 (Supplementary Figure 1C,D) cross‐react with cardiac myosin (Figure 4O), laminin (Figure 4P), tropomyosin (Figure 4Q), and keratin (Figure 4R). No cross‐reactivity with tissue proteins was observed in sera from rats injected with PBS/CFA/0.6 µg BPTx and GAS rM5/CFA without BPTx. Significantly higher levels of cross‐reactive antibodies were observed in rats injected with GAS rM5/CFA/0.3 µg and 0.6 µg BPTx. Although high antibody levels were observed in rats injected with GAS rM5/CFA/0.3 µg BPTx, none of these rats showed functional ECG changes, unlike the rats injected with GAS rM5/CFA/0.6 µg BPTx. Collectively, these observations highlight the need for a strong inflammatory response in addition to the presence of autoantibodies to cause pathology.

### 3.5 BPTx Promotes Infiltration of Inflammatory Cells

**Figure 5:**
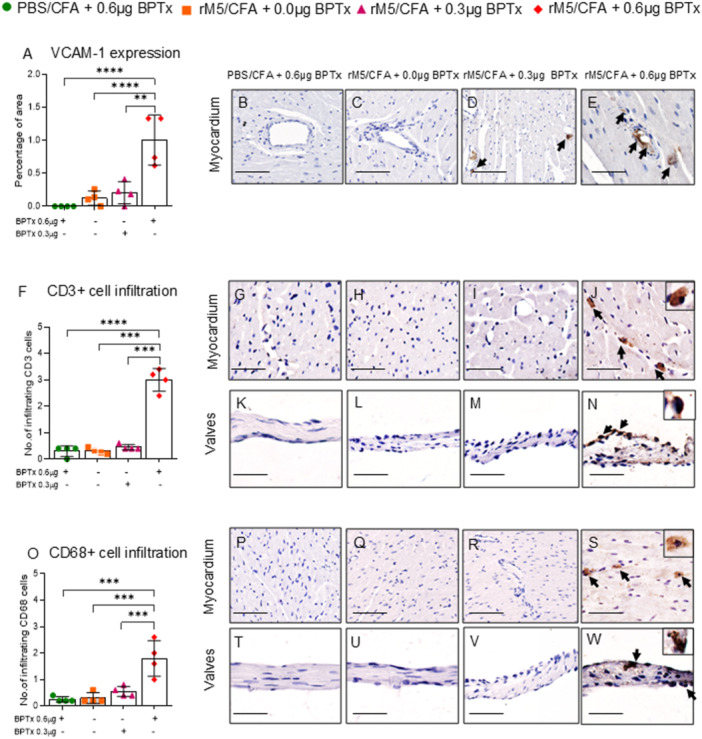
BPTx induces increased expression of adhesion molecules and infiltration of mononuclear cells into the cardiac tissue. Myocardial tissue was stained to detect expression of the endothelial cell adhesion molecule VCAM‐1. Percentage of VCAM‐1 expression in the myocardium (A). Representative images of myocardium highlighting increased expression of VCAM‐1 (B‐E) in rats injected with GAS rM5/CFA/0.6 µg BPTx (E) compared to rats injected with GAS rM5/CFA/0.3 µg BPTx (D) and GAS rM5/CFA alone (C) and PBS/CFA/0.6 µg of BPTx (B). T‐cells and macrophages in the mononuclear cells infiltrate were characterized by staining with pan T‐cell antibody against CD3^+^ and CD68^+^ respectively (F and O). A significantly higher infiltration of CD3^+^ T‐cells and CD68^+^ macrophages were observed in myocardium and valvular tissues of rats injected with GAS rM5/CFA/0.6 µg BPTx (J, N, S, W) compared to rats injected with GAS rM5/CFA/0.3 µg BPTx (I, M, R, V) and GAS rM5/CFA alone (H, L, Q, U) and PBS/CFA/0.6 µg BPTx (G, K, P, T). Images magnified at ×200, snippets magnified at ×400, and scale bar represents 50 µm. Data are from single experiment with age‐matched rats injected with GAS rM5 (*n* = 4). Statistics: mean ± SEM (*n* = 4); (A, F and O) *t* test; **p < 0.01 and ***p < 0.001.

In ARF/RHD cardiac inflammation is characterized by infiltration of circulating leukocytes into the myocardial and valvular tissue. In addition, inflammatory cytokines and GAS antibodies up‐regulate vascular cell adhesion molecules (VCAM)‐1 expressed on endothelial cells and promote infiltration of mononuclear cells into the myocardium and valvular tissues [14, 40]. To determine whether BPTx induces cell adhesion that could lead to infiltration of inflammatory cells, we assessed the level of VCAM‐1 expression in myocardium. VCAM‐1 expression was higher in rats injected with GAS rM5/0.6 µg BPTx compared to rats injected with PBS/CFA/0.6 µg BPTx, GAS rM5 without BPTx, and GAS rM5/CFA/0.3 µg BPTx (Figure 5A–E). Furthermore, increased infiltration of CD3^+^ T‐cells (Figure 5J,N) and CD68^+^ macrophages (Figure 5S,W) into the myocardium and valvular tissue was observed in rats injected with GAS rM5/CFA/0.6 µg BPTx. No significant difference in infiltration of CD3^+^ T‐cells and CD68^+^ macrophages were observed in rats injected with PBS/CFA/0.6 µg BPTx, GAS rM5 without BPTx and GAS rM5/CFA/0.3 µg BPTx (Figure 5G–M,P–V).

### 3.6 Pertussis Toxin Increases Infiltration of IFN‐Γ^+^ and IL‐17A^+^ Producing T‐Cells Into Myocardium and Valvular Tissues

**Figure 6:**
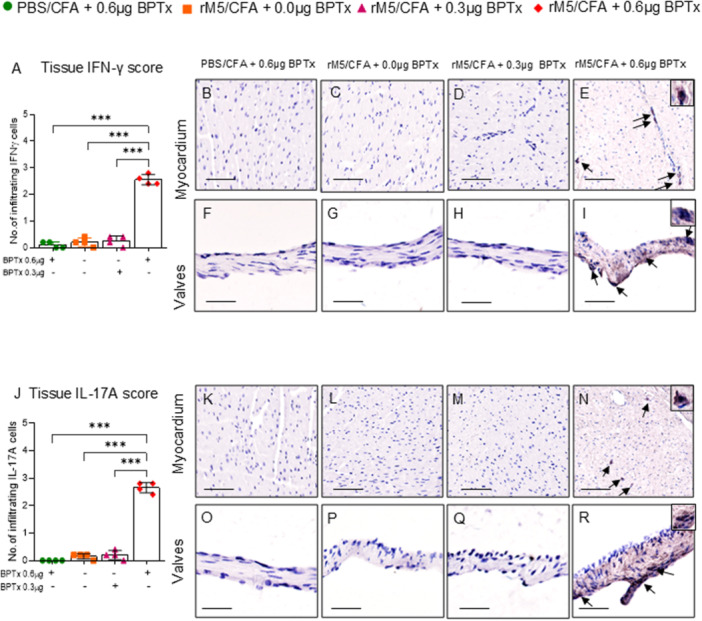
BPTx induces infiltration of IFN‐γ^+^ and IL‐17A^+^ T‐cells into the cardiac tissue. Cumulative number of infiltrating IFN‐γ^+^ (A) and IL‐17A^+^ T‐cells (J) in myocardial and valvular tissue. Significantly higher levels of IFN‐γ^+^ and IL‐17A^+^ cells were observed in and representative images of IFN‐γ^+^ and IL‐17A^+^ T‐cells in myocardial and valvular tissue (I and R). A significantly higher infiltration of IFN‐γ^+^ and IL‐17A^+^ T‐cells were observed in the myocardium and valvular tissues of rats injected with GAS rM5/CFA/0.6 µg BPTx (E, I, N, R) compared to rats injected with GAS rM5/CFA/0.3 µg BPTx (D, H, M, Q) and GAS rM5/CFA alone (C, G L, P) or PBS/CFA/0.6 µg BPTx (B, F, K, O). Images magnified at ×200, snippets magnified at ×400, and scale bar represents 50 µm. Data are from single experiment with age‐matched rats injected with GAS rM5 (*n* = 4). Statistics: mean ± SEM (*n* = 4); (A and J) *t* test; **p < 0.01 and ***p < 0.001.

We observed an increase in the infiltration of IFN‐γ^+^ (Figure 6E,I) and IL‐17A^+^ producing cells into the myocardium and valvular tissue of rats injected with GAS rM5/CFA/0.6 µg BPTx compared to rats injected with PBS/0.6 µg BPTx, GAS rM5 without BPTx, and GAS rM5/CFA/0.3 µg BPTx (Figure 6N,R). Moreover, when we quantified the tissue‐infiltrating cells expressing IFN‐γ^+^ and IL‐17A^+^, we found that rats injected with GAS rM5/CFA/0.6 µg BPTx had significantly higher numbers of IFN‐γ^+^ and IL‐17A^+^ producing cells infiltrating into the myocardium (Figure 6I) and valvular tissue compared to other groups (Figure 6J).

## 4 Discussion

ARF/RHD is an autoimmune disorder triggered by inflammatory signals induced by preceding GAS infection [3, 41]. Both preclinical and clinical evidence also suggest the involvement of Transforming growth factor β1 (TGF‐β1) signalling and other fibro‐inflammatory pathways, in initiation and progression of the disease [42]. Therefore, the initiation and progression of ARF/RHD may involve the dysregulation of inflammatory cascades that emerge following every bout of GAS infections that has the potential to increase the level of cross‐reactive antibodies and T‐cells. In this study, we demonstrate intraperitoneal administration of BPTx induces robust inflammatory responses that promote the development of autoimmune myocarditis and valvulitis in the RAV model. We have now mirrored the clinical scenario in the RAV model and demonstrate that a strong inflammatory response is required to induce cardiac damage in the presence of GAS M protein.

Initially, we assessed the inflammatory responses induced by BPTx and Bordetella *pertussis* toxoid (inactivated whole cell antigens extracts of *Bordetella pertussis*; BPToxoid) following subcutaneous injection of GAS rM5/PBS emulsified in CFA. Rats administered BPTx showed a robust IFN‐γ^+^ and IL‐17A^+^ response leading to functional and inflammatory changes in cardiac tissue compared to rats administered BPToxoid. It has been previously reported that BPTx acts as a T‐cell mitogen [43, 44] and induces Th1 and possibly Th17 responses, enhancing autoimmune pathology in EAE and EAU. Similarly, our results provide direct evidence that administration of BPTx early after injection of GAS rM5 exacerbates inflammation and induces cardiac pathology in the RAV model. The imbalance between effector T cells (Th1/Th17) and regulatory T cells (Treg) play a very important role in autoimmune pathogenesis including ARF/RHD [45]. However, the precise mechanisms of how BPTx that leads to Th1/Treg imbalance are yet to be characterised. In this study the autoimmune pathology is exacerbated by BPTx due to the upregulation of effector T cells (Th1/Th17) responses. Expanding on this knowledge, future studies will also seek to investigate the role of Tregs during the initial stages of the disease process.

BPTx plays a key role in inducing inflammatory cytokines in various experimental models [28, 34, 46]. It has been demonstrated that administration of BPTx enhanced the production of IL‐12 and TNF‐α thereby facilitating the development of Th1 responses [47] and also promoting the generation of IL‐17A^+^ producing CD4^+^ T‐cells. BPTx induces the maturation of dendritic cells [48, 49, 50] and leads to expansion of effector T‐cells and secretion of IFN‐γ [51]. Furthermore, recent studies have demonstrated that BPTx induces IL‐1β secretion by myeloid cells, which is required to prime auto‐reactive Th1 and Th17‐cells [34, 35]. BPTx has also been shown to directly activate macrophages and dendritic cells that present antigens to autoreactive T‐cells secreting the inflammatory cytokine, IL‐6 [48]. Additionally, studies in other animal models have demonstrated that BPTx enhances the release of IL‐6 and mediates Th‐17 differentiation [33, 52]. IL‐6 is an important inflammatory cytokine that promotes an acute phase response to infections and has been implicated in the pathogenesis of multiple autoimmune diseases including ARF/RHD [3, 4, 53]. Moreover, IL‐6 has pleiotropic activity on a broad range of immune cells. It promotes B‐cell activation and antibody responses, and T‐cell activation and differentiation [54, 55]. Therefore, the elevated levels of IL‐1β and IL‐6 observed in this study may play a major role in potentiating the autoimmune response following the injection of GAS rM5/CFA/0.6 µg BPTx. This study is consistent with our previous observations where repetitive injection of GAS rM5 induced an efficient Th1/Th17 response required for the initiation and exacerbation of cardiac changes observed in the RAV model [11, 12, 17, 20].

In active EAE models, BPTx acts as an immune adjuvant and leads to a compromised blood–brain barrier allowing inflammatory cells to enter the brain [56, 57]. It has been proposed that BPTx alters the vascular permeability and thereby facilitates the breakdown of blood–tissue barriers and promotes transmigration of inflammatory cells into the target organ [58, 59]. What's more, studies have shown that BPTx increases the expression of adhesion molecules that initiate leukocyte infiltration into the brain [60]. Whether a similar mechanism is at play in the RAV model, which has been shown to develop neurobehavioral changes akin to Sydenham's chorea, remains to be determined.

It was previously shown that T‐cell cytokines induce inflammatory responses and play a major role in the pathogenesis of ARF/RHD [3, 41, 61, 62, 63]. Therefore, we analyzed peripheral blood levels and tissue infiltration of IFN‐γ^+^ and IL‐17A^+^ producing cells following injection of GAS rM5 and different doses of BPTx. The autoreactive antibodies along with Th1/Th17 cells contribute to the development and progression of ARF/RHD [45, 64, 65, 66]. In our study, we found that BPTx increased the infiltration of CD3^+^ T‐cells and CD68^+^ macrophages into the myocardium and valvular tissues. These heart infiltrating T‐cells in the RAV model expressed IFN‐γ^+^ and IL‐17A^+^. In addition, it has been found that the expression of VCAM‐1 on rat endothelial cells facilitate the infiltration of T‐cells [14]. Inflammatory cytokines have also been shown to mediate and prime GAS rM5 induced cardiac pathology. We therefore assessed the levels of inflammatory cytokines, IFN‐γ and IL‐17A, in peripheral circulation and infiltration of IFN‐γ^+^ and IL‐17A^+^ secreting cells into myocardium and valvular tissues. We found that BPTx increased infiltration of IFN‐γ^+^ and IL‐17A^+^ secreting mononuclear cells into cardiac tissue, which may play a major role in driving the pathological process. These cellular changes occurring within the heart valves, in combination with haemodynamic stress and the transvascular pressure gradient, may result in preferential damage of the mitral valve [42]. Recent studies have also demonstrated that activation of TGF‐β1/Smad signalling pathway promotes cell adhesion to the extracellular matrix of cardiac tissue and induces cardiac fibrosis in RHD [42].

In experimental models, adjuvants play an important role in promoting both antibody and T‐cell responses that lead to pathology. In this study, we have demonstrated that the BPTx induced IFN‐γ^+^ and IL‐17A^+^ T‐cell responses are essential for the development of carditis. We first found that administration of BPTx on day 1 and 3 of primary injection of GAS rM5/CFA enhances the production of cross‐reactive antibodies against cardiac tissue proteins. Second, we found that the BPTx treatment induced a robust Th1/Th17 response that accelerated the inflammatory response triggered by GAS rM5, exacerbating cardiac damage in the RAV model. However, in this study, we have not evaluated the specific mechanisms by which BPTx drives the inflammatory response induced by GAS rM5. While IFN‐γ and IL‐17A responses were evaluated, the study did not investigate the role of regulatory T cells and other inflammatory mediators that may be crucial in disease modulation. Elucidating the specific mechanism underlying this phenomenon requires further investigations using techniques that can simultaneously assess multiple inflammatory parameters in preclinical models [67]. Furthermore, including Echocardiography [12, 13] pre and post injection of BPTx, would provide an insight into the structural and functional changes occurring during the disease process. This would be informative for the model as BTx is a toxin derived from a Gram‐negative organism and may not mimic the changes caused by repetitive infections with Gram‐positive GAS, as observed in ARF/RHD. Further investigation of other GAS‐associated molecules, particularly superantigens, is warranted as they may serve as triggers that potentiate GAS rM5‐induced inflammation leading to cardiac damage.

## 5 Conclusion

This study demonstrates that the combined use of CFA/BPTx is far superior in triggering an immune response required to cause significant cardiac tissue damage following exposure to GAS rM5. Therefore, in susceptible individuals GAS infection initiates an inflammatory autoimmune response that leads to ARF, and multiple episodes of subsequent GAS infection accelerate cardiac damage leading to RHD. The RAV model has already made an important contribution in assessing anti‐GAS vaccine safety in preclinical trials [24]. Furthermore, the potential to identify early biomarkers [68] of disease progression, and to test potential therapeutics to downregulate inflammatory responses can be addressed using this model.

## Author Contributions

**Rukshan A. M. Rafeek:** conceptualization, data curation, formal analysis, investigation, methodology, resources, software, validation, visualization, writing – original draft, writing – review and editing. **Simone L. Reynolds:** conceptualization, investigation, methodology, writing – review and editing. **Manisha Pandey:** conceptualization, investigation, writing – review and editing. **David J. McMillan:** conceptualization, methodology, resources, writing – review and editing. **Kadaba S. Sriprakash:** conceptualization, methodology, writing – review and editing. **Michael F. Good:** conceptualization, investigation, methodology, supervision, writing – review and editing. **Natkunam Ketheesan:** conceptualization, funding acquisition, investigation, methodology, project administration, resources, supervision, validation, writing – review and editing.

## Ethics Statement

All experimental protocols involving animals were approved by the Animal Ethics Committee of University of New England (UNE) (AEC 19‐013/ARA22‐065). All procedures involving animals were in accordance with Australian code for the care and use of animals for scientific purposes and followed the ARRIVE guidelines.

## Conflicts of Interest

The authors declare no conflicts of interest.

## Supporting information

Fig. S1 shows the detailed experimental procedure including treatment strategy and techniques used to assess the inflammatory responses (A) and antibody reactivity against BPTx and BPToxoid.

Fig. S2 shows the gating strategy used in FACS assay.

Fig. S3 shows scoring system for cardiac tissue to assess the inflammatory cell infiltration.

Rafeek et al 2024 ARRIVE guideline checklist ‐ E10 only.

Supplementary Table 1.

## Data Availability

The data underlying this article are available in the article and in its online supporting information.
