# Supplementary figures and images for: Inflammatory Responses Potentiate GAS M Protein Induced Cardiac Damage in an Experimental Model of Rheumatic Heart Disease
Source: Immun Inflamm Dis. 2025 Jul 11;13(7):e70221. doi: 10.1002/iid3.70221 (PMC12246833; doi:10.1002/iid3.70221)

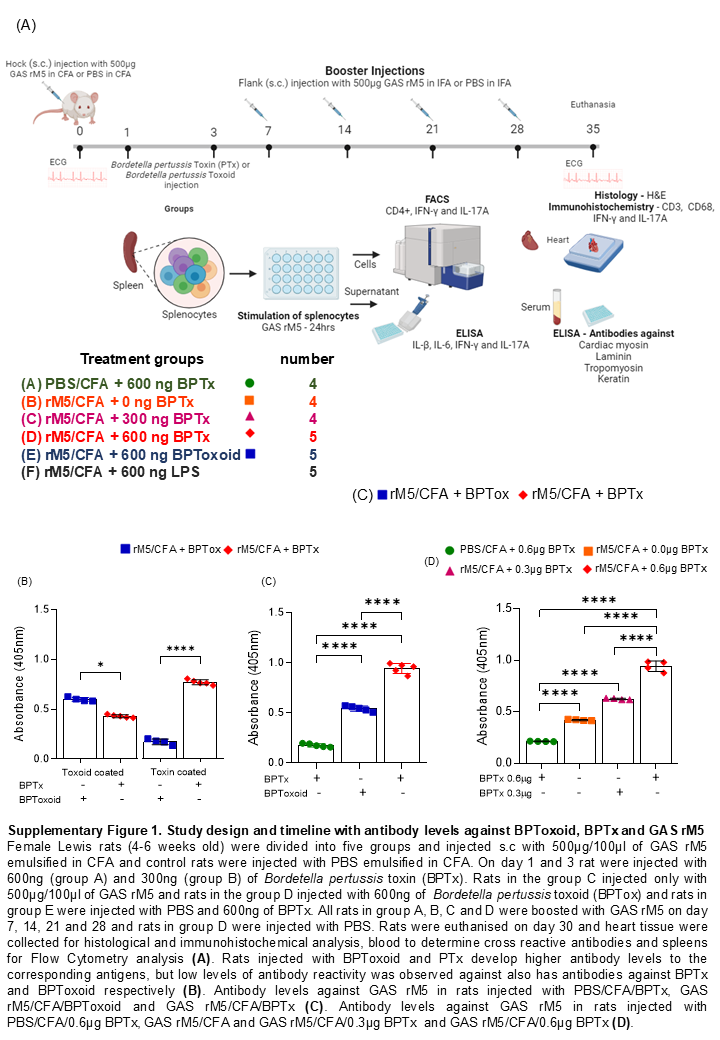

Supplement: Supplementary file 1 — Fig. S1 shows the detailed experimental procedure including treatment strategy and techniques used to assess the inflammatory responses (A) and antibody reactivity against BPTx and BPToxoid. [file IID3-13-e70221-s002.tif]

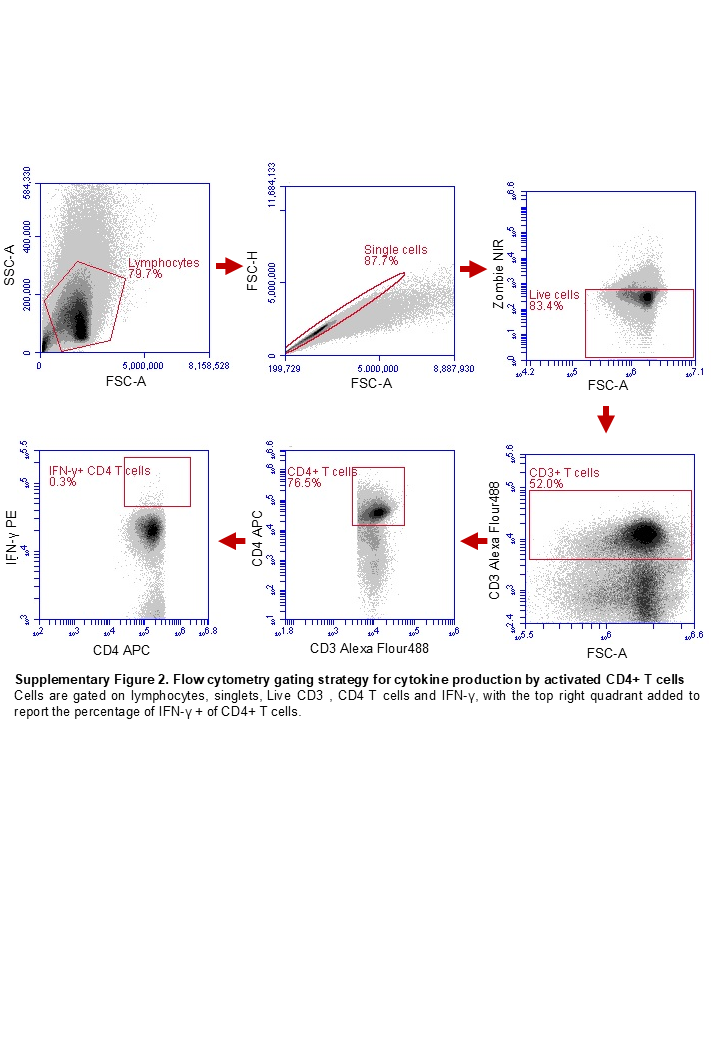

Supplement: Supplementary file 2 — Fig. S2 shows the gating strategy used in FACS assay. [file IID3-13-e70221-s005.tif]

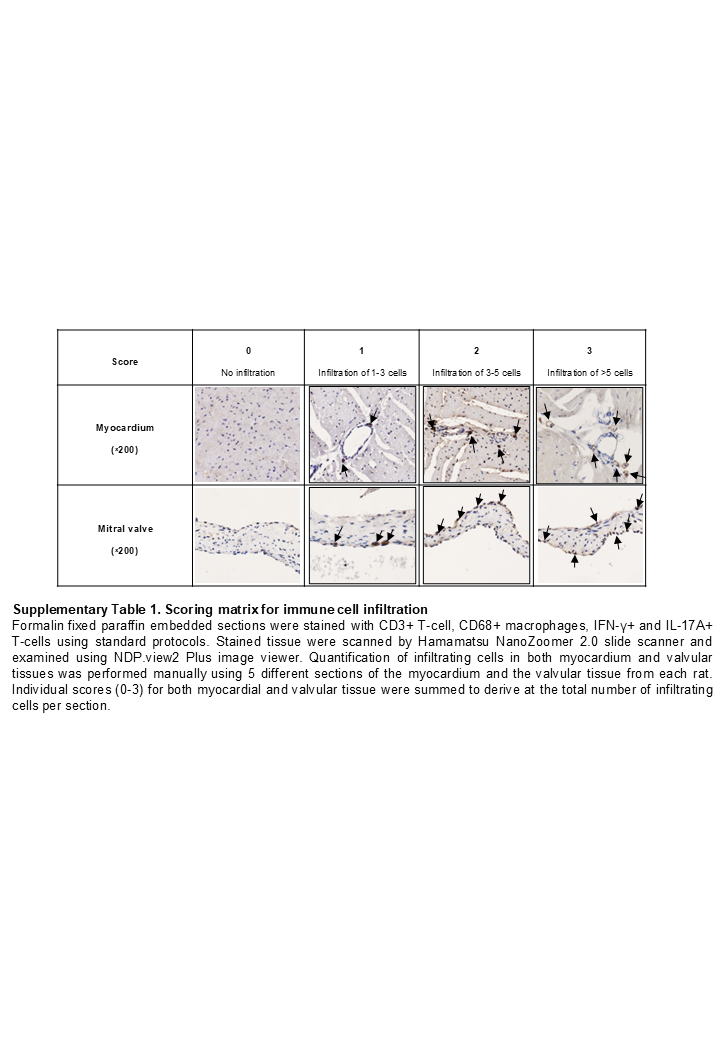

Supplement: Supplementary file 3 — Fig. S3 shows scoring system for cardiac tissue to assess the inflammatory cell infiltration. [file IID3-13-e70221-s003.tif]
