# Supplementary material for: Inflammatory Responses Potentiate GAS M Protein Induced Cardiac Damage in an Experimental Model of Rheumatic Heart Disease
Source: Immun Inflamm Dis. 2025 Jul 11;13(7):e70221. doi: 10.1002/iid3.70221 (PMC12246833; doi:10.1002/iid3.70221)
Supplement: Supplementary file 5 — Supplementary Table 1. [file IID3-13-e70221-s001.pptx]

## Slide 1
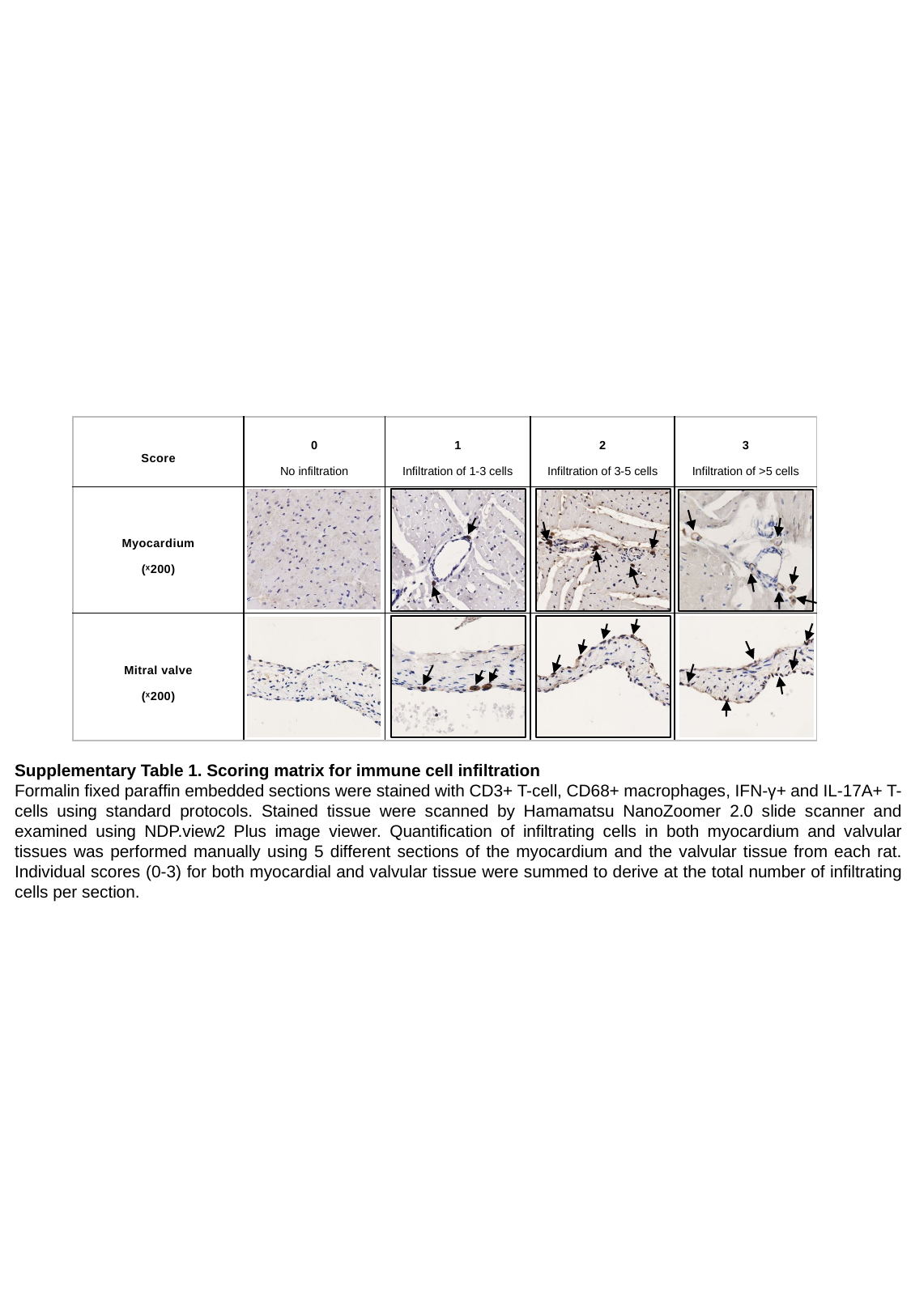

| Score | 0 No infiltration | 1 Infiltration of 1-3 cells | 2 Infiltration of 3-5 cells | 3 Infiltration of >5 cells |
| --- | --- | --- | --- | --- |
| Myocardium (ˣ200) | | | | |
| Mitral valve (ˣ200) | | | | |
Supplementary Table 1. Scoring matrix for immune cell infiltration
Formalin fixed paraffin embedded sections were stained with CD3+ T-cell, CD68+ macrophages, IFN-γ+ and IL-17A+ T-cells using standard protocols. Stained tissue were scanned by Hamamatsu NanoZoomer 2.0 slide scanner and examined using NDP.view2 Plus image viewer. Quantification of infiltrating cells in both myocardium and valvular tissues was performed manually using 5 different sections of the myocardium and the valvular tissue from each rat. Individual scores (0-3) for both myocardial and valvular tissue were summed to derive at the total number of infiltrating cells per section.
